# Identification of the first large deletion in the *CLDN16* gene in a patient with FHHNC and late-onset of chronic kidney disease: case report

**DOI:** 10.1186/s12882-015-0079-4

**Published:** 2015-07-02

**Authors:** Paulo Marcio Yamaguti, Pollyanna Almeida Costa dos Santos, Bruno Sakamoto Leal, Viviane Brandão Bandeira de Mello Santana, Juliana Forte Mazzeu, Ana Carolina Acevedo, Francisco de Assis Rocha Neves

**Affiliations:** University Hospital of Brasilia, University of Brasilia, Brasilia, Brazil; Laboratory of Oral Histopathology, Faculty of Health Sciences, University of Brasilia, Brasilia, Brazil; Faculty of Medicine, University of Brasilia, Brasilia, Brazil; Nephrology Division, Hospital de Base de Brasilia, Soclimed Nephrology and Dialysis Unit, Brasilia, Brazil; Laboratory of Genetics, Faculty of Medicine, Faculty of Health Sciences, University of Brasilia, Brasilia, Brazil; Laboratory of Oral Histopathology, Faculty of Health Sciences, University of Brasilia, Brasilia, Brazil; Soclimed Nephrology and Dialysis Unit, Brasilia, Brazil; Laboratório de Farmacologia Molecular, Faculdade de Ciências da Saúde, Universidade de Brasília, Campus Universitário Darcy Ribeiro, Brasília, Brazil

**Keywords:** *CLDN16*, Claudin-16, Hypercalciuria, Hypomagnesemia, Nephrocalcinosis

## Abstract

**Background:**

Familial hypomagnesemia with hypercalciuria and nephrocalcinosis is a rare autosomal recessive renal disease characterized by tubular disorders at the thick ascending limb of Henle’s loop. It is caused by mutations in the tight junction structural proteins claudin-16 or claudin-19, which are encoded by the *CLDN16* and *CLDN19* genes, respectively. Patients exhibit excessive wasting of calcium and magnesium, nephrocalcinosis, chronic kidney disease, and early progression to end-stage renal failure during infancy.

**Case presentation:**

We here report the phenotype and molecular analysis of a female Brazilian patient with a novel large homozygous deletion in the *CLDN16* gene. The proband, born from consanguineous parents, presented the first symptoms at age 20. Clinical examination revealed hypocalcemia, hypomagnesemia, nephrocalcinosis, mild myopia, high serum levels of uric acid and intact parathyroid hormone, and moderate chronic kidney disease (stage 3). She and her mother were subjected to *CLDN16* and *CLDN19* mutational analysis. In addition, the multiplex ligation-dependent probe amplification method was used to confirm a *CLDN16* multi-exon deletion. Direct sequencing revealed a normal *CLDN19* sequence and suggested a large deletion in the *CLDN16* gene. Multiplex ligation-dependent probe amplification showed a homozygous *CLDN16* multi-exon deletion (E2_E5del). The patient initiated conventional treatment for familial hypomagnesemia with hypercalciuria and nephrocalcinosis and progressed to end-stage kidney disease after five years.

**Conclusions:**

This study provides the first report of a large homozygous deletion in the *CLDN16* gene causing familial hypomagnesemia with hypercalciuria and nephrocalcinosis with late onset of the first symptoms. This description expands the phenotypic and genotypic characterization of the disease. The late-onset chronic kidney disease in the presence of a homozygous deletion in the *CLDN16* gene reinforces the great variability of genotype-phenotype manifestation in patients with familial hypomagnesemia with hypercalciuria and nephrocalcinosis.

## Background

Familial hypomagnesemia with hypercalciuria and nephrocalcinosis (FHHNC) is a rare autosomal recessively inherited tubulopathy that is characterized by excessive calcium and magnesium renal excretion. FHHNC patients harbor homozygous or compound heterozygous mutations in the *CLDN16* (OMIM248250) or *CLDN19* genes (OMIM248190), which encode the tight junction structural proteins claudin-16 [1] and claudin-19 [2], respectively.

Claudin-16 and −19 belong to a large protein family comprising at least 27 different isoforms in mammals. Claudin-16 expression is restricted to the medullar and cortical thick ascending limb of the loop of Henle (TAL) and the distal convoluted tubule [3]. Claudin-19 co-localizes with claudin-16 in the kidney and is expressed in the retinal epithelium [2] and in myelinated peripheral neurons [4]. At the TAL, both claudin-16 and claudin-19 *cis*-interact to form a selective paracellular cation channel complex [5, 6]. Although different studies have demonstrated varying roles of each protein in the complex [5, 7, 8], it is currently well accepted that the claudins interact to play a pivotal role in the generation of transepithelial NaCl diffusion potential in the tubular lumen, which drives the paracellular force for magnesium and calcium reabsorption [9, 10].

The onset of the first symptoms of FHHNC often occurs during infancy and is characterized by recurrent urinary tract infections, nephrolithiasis, polyuria, polydipsia, and, in some cases, tetanic convulsions. Biochemical findings include elevated serum intact parathyroid hormone levels, incomplete distal renal tubular acidosis, hypocitraturia, hyperuricemia, and an impaired glomerular filtration rate [1, 2, 11, 12]. In addition, patients with *CLDN19* mutations usually present severe ocular abnormalities such as macular coloboma, nystagmus, retinopathy, and visual loss [2, 12–14]. In patients with *CLDN16* mutations, milder visual disturbances such as myopia, astigmatism, hypermetropia, and/or strabismus have been described [1, 11].

To date, about 50 different mutations have been reported in the *CLDN16* gene (40 missense/nonsense, 5 splicing, 3 small deletions, and 2 small indels), and 15 mutations have been reported in the *CLDN19* gene (13 missense/nonsense, 1 small deletion, and 1 large deletion) (http://www.hgmd.cf.ac.uk). Functional studies have demonstrated that *CLDN16* mutations may induce partial or complete loss of protein function [12, 15]. Usually, mutations that result in complete loss of function of both alleles induce a phenotype with earlier onset of the symptoms and a more rapid decline of the glomerular filtration rate [12, 15]. By contrast, mutations resulting in partial loss of function seem to display a slower progression to end-stage renal disease. However, in some cases, no genotype-phenotype correlation was observed [1, 16, 17].

In the present study, we describe, for the first time, an FHHNC patient who presented a multi-exon deletion in the *CLDN16* gene and late onset of symptoms. This description opens new avenues for understanding the importance of claudin-16 in calcium and magnesium homeostasis in humans, as well as its role in disease.

## Case presentation

### Clinical characteristics

The proband was born from consanguineous parents (first-degree cousins). She was the only daughter of a Brazilian Caucasian couple. The father was reported to be healthy but he could not be examined because he was living in another city. The mother was also healthy and did not have a medical history of nephrolithiasis or urinary infection. Her plasmatic and urinary biochemical analysis presented normal values, and the ultrasound did not reveal any evidence of renal disease.

The proband presented normal development and growth and reported to be healthy until the age of 20 years, when she started to complain of frequent abdominal and joint pain. Serum and urinary biochemical exams revealed hypercalciuria, hypomagnesemia, and increased levels of triglycerides, uric acid, urea, and intact parathyroid hormone, with moderate chronic kidney disease (CKD), diagnosed at stage 3 (Table 1). Her serum calcium level was also under the lower limit. The fractional excretions of calcium and magnesium were high, suggesting FHHNC (Table 1). Although there was no medical history of nephrolithiasis or recurrent urinary tract infections, nephrocalcinosis was detected under renal ultrasound. Moreover, bone densitometry demonstrated osteoporosis in the femur. Ophthalmic exams revealed only mild myopia.

**Table 1:** Biochemical serum and urine analysis of the proband at the age of diagnosis (20 years old) through to the age when she began peritoneal dialysis (25 years old)

Exam (reference values)	Age (years)					
	20	21	22	23	24	25
Serum calcium (8.3–10.6 mg/dL)	7.1	8.3	9.1	7.9	7.5	10.9
Serum magnesium (1.3–2.7 mg/dL)	0.9	1.3	1.2	1	0.9	0.9
Uric Acid (2.6–6.0 mg/dL)	7.5	7.5	11.9	11.8	12.2	13.1
Creatinine (0.50–1.10 mg/dL)	1.9	2.1	2.4	3.7	4.8	5.6
iPTH (12–72 pg/mL)	120	321	305	542	900	2620
Urea (19–49 mg/dL)	72	84	85	84	137	220
FECa (<0.20)	3.78	4.24	3.53	3.03	2.5	2.7
FEMg (3–5)	14.6	15.8	19.1	28.9	20.4	19.3
Urinary pH (4.8–7.0)	7	7	6.5	6	5	6.5
Glomerular Filtration Rate (81–134 mL/min 1.73 m^2^)	51	45	34	33	25	11

At the age of diagnosis, the patient presented high levels of uric acid, urea, and intact parathyroid hormone. At age 20, she presented stage 3, moderate chronic kidney disease (CKD). She presented high fractional excretion of calcium and magnesium. In six years, she progressed to end-stage kidney disease. Formulas: FECa (Ca(U) X Cr(S)/Ca(S) X Cr(U) X 100); FEMg (Mg(U) X Cr(S)/Mg(S) X Cr(U) X 100); Glomerular Filtration Rate: [140 – age (years) X weight (kg)/72 X Cr(S) (mg/dL) X 0.85 (female)]. Abbreviations: iPTH, intact parathyroid hormone; FECa, fractional excretion of calcium relative to creatinine; FEMg, fractional excretion of magnesium relative to creatinine; Ca, calcium; Mg, magnesium; U, urinary; S, serum; Cr, creatinine

When diagnosed, the patient began treatment with magnesium citrate (1 g/day), hydroclorotiazide (25 mg/day), calcitriol (0.25 μg/day), calcium carbonate (1 g/day), alopurinol (300 mg/day), and followed the recommendation of high ingestion of water (at least 3 L/day). In the following years, the dosages were slightly changed according to the disease progression. The patient followed all the recommendations and adhered to the proposed treatment, but after five years her renal function rapidly progressed to end-stage kidney disease (glomerular filtration rate decreased from 51 to 11 mL/min 1.73 m^2^) and she initiated peritoneal dialysis at the age of 26.

The proband and her mother signed the informed consent form, in accordance with the ethical standards of the responsible national committee on human experimentation (CONEP 1440/2001) and the Helsinki Declaration. They were subjected to physical examination, including ophthalmic exams, and serum and urine biochemistry analysis. The diagnosis of nephrocalcinosis was made based on results of renal ultrasonography. Ophthalmic exams were conducted to determine the presence of myopia, pigmentary retinitis, macular coloboma, strabismus, astigmatism, and/or nystagmus.

### Molecular analysis and multiplex ligation-dependent probe amplification (MLPA)

For molecular analysis, venous blood was collected and genomic DNA was isolated from leukocytes using Wizard Genomic DNA Purification Kit (Promega; Madison, WI, USA). DNA fragments of the human *CLDN16* and *CLDN19* gene-coding regions and exon-intron boundaries were amplified using gene-specific primer sets (*CLDN16* primers sets had been previously described by Simon *et al*. [3] and *CLDN19* primers sequences were kindly provided by Dr. Martin Konrad/ University Children’s Hospital, Munster, Germany). Polymerase chain reaction (PCR) products were purified and subject to bidirectional DNA sequencing reactions using the BigDye3.1 terminator in an ABI3730 sequencer (ABI Prism) at a genomic core facility (Macrogen Inc.; Seoul, Korea). Sequencing results were compared to the *CLDN16* and *CLDN19* reference sequences available at ENSEMBL (www.ensembl.org).

After conventional PCR and sequencing of the *CLDN16* and *CLDN19* genes, synthetic MLPA probes were designed to confirm the suspicion of a *CLDN16* partial deletion. The target sequence of each synthetic half probe was designed according to the specifications described by Stern et al. [18]. Probe pairs for exons 3 and 5 of the *CLDN16* gene were designed. Two control probe pairs for the *VIPR2* and *KIAA0056* genes were included in the probe set. MLPA reactions were performed following the standard protocol (MRC-Holland protocol; http://www.mlpa.com). Trace data were analyzed using the Gene Mapper v4.0 software (Applied Biosystems; Foster City, CA, USA), and the integrated peak areas and heights were exported to an Excel spreadsheet (Microsoft; Silicon Valley, CA, USA). For each sample, the peak heights were first normalized to the average peak height of the control probes, followed by normalization to the average peak height of control samples, obtained from healthy and non-related patients, included in the run. The sample run was considered acceptable if the ratio to the control probe pairs was between 0.8 and 1.2. The threshold value for deletion was set to 0.75.

Direct sequencing of the *CLDN19* coding regions of the proband did not reveal any mutations or single nucleotide polymorphisms. In the *CLDN16* gene, exon 1 and the promoter region were amplified and presented the normal sequences. However, after several attempts, it was not possible to amplify this gene from exon 2 through exon 5, suggesting a homozygous gene deletion.

**Fig. 1:**
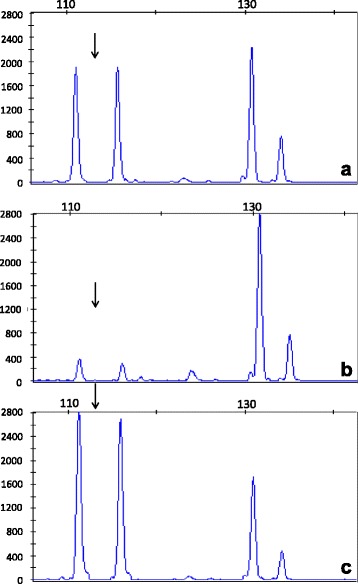
Pherograms corresponding to the electrophoresis of the multiplex ligation-dependent probe amplification assay (**a**: mother; **b**: proband; **c**: control sample). The first two peaks (arrows) represent annealing of the *CLDN16* probes to the genomic DNA. The last two peaks are the two control probes. Notice the two first peaks in the control sample (**c**), the absence of these peaks in the proband (**b**), and half of the peak height in the mother (**a**). The control probes remained constant in all samples

Under MLPA analysis, the probes designed for exons 3 and 5 presented half the expected signal in the mother. In the proband, there was no amplification, thus suggesting a homozygous gene deletion (Fig. 1). The control genes could be normally amplified (Fig. 1).

### Claudin and FHHNC

Claudin proteins contain four domains: a short intracellular N-terminus, four transmembrane domains, two extracellular loops, and a long C-terminal cytosolic tail. The first extracellular loop appears to line the paracellular pore and determines the protein’s selectivity, whereas the second extracellular loop mediates *trans* interactions [19]. The C-terminal cytosolic tail plays roles in protein trafficking to the tight junction and in protein stability [19]. To date, about 50 different *CLDN16* mutations have been identified in FHHNC patients in the coding regions of all four domains (http://www.hgmd.cf.ac.uk). The present case represents the first report of a patient with a large deletion from exon 2 to 5 of *CLDN16*, which likely induced complete loss of protein function.

Functional assays have revealed that different mutant proteins can display normal trafficking to the cell membrane or remain in the cell, localized either to lysosomes or to the endoplasmic reticulum. Moreover, even when they are properly targeted to the tight junctions, some claudin-16 mutants (p.L145P, p.L151F, p.G191R, p.A209T, and p.F232C) fail to interact with claudin-19, thereby impairing the synergistic effect of the complex. This results in partial or complete disruption of calcium and magnesium homeostasis [5, 15, 20]. The heterodimeric claudin-16 and claudin-19 interaction is reported to be essential for the divalent cations selectivity of the paracellular channels at the TAL [5, 6]. Other studies using RNA interference showed that all mutations that disrupted this interaction abrogated the function of the whole complex [21]. Experimental animal models demonstrated that the silencing of either the *Cldn16* or *Cldn19* gene resulted in the absence of the other protein (claudin-19 or claudin-16, respectively) at the tight junctions in the TAL [6].

To date, it is not possible to predict which of the two proteins is more important for calcium and magnesium homeostasis. In 2012, Godron *et al.* compared the renal progression of patients with *CLDN16* and *CLDN19* mutations, and observed that patients with *CLDN19* mutations showed a more severe decline. However, they assumed that the mutation types in this cohort of patients were more severe in the *CLDN19* group, including a large deletion and frameshift mutations [12].

In most cases, the median age of FHHNC onset and diagnosis is during infancy. In 2008, Konrad *et al.* evaluated 23 patients with different *CLDN16* mutations that induced a complete loss of function [15]. They observed that the median age for the onset of the first symptoms was 2.2 years; approximately 30 % of these patients presented CKD at diagnosis, and 50 % required renal replacement therapy by age 15 [15]. Other studies comprising FHHNC patients with complete loss of function of claudin-16 reported the median age of the first symptoms ranging from 0.1 to 7 years, and the age of clinical diagnosis ranging from 0.5 to 12 years [1, 12, 22, 23]. Our patient presented the first symptoms only at 20 years of age, and the biochemical exams revealed moderate CKD. Her renal function was surely altered before diagnosis, but she had no previous history of urinary infection or any symptom of renal disease.

This is the first report of FHHNC caused by a large deletion in the *CLDN16* gene. To date, there is only one case report of a large deletion in the *CLDN19* gene, which was detected in a Tunisian family. However, the individual phenotypes of the patients were not described [12]; therefore, we could not compare our results with these patients with respect to the disease progress and renal phenotype. Although we have not performed functional assays, we hypothesize that this multi-exon deletion observed in our patient induced a complete loss of function of claudin-16, which consequently affected the function of the whole heterodimer.

In 2010, a study using *Cldn16* knockout mice yielded interesting results concerning the age of onset of hypomagnesemia. Neonatal animals exhibited normomagnesemia, while juvenile animals presented only mild hypomagnesemia [9]. The late onset of hypomagnesemia was triggered by a further decrease in claudin-19 levels during development. Claudin-19 expression decreased with age (30 % lower expression in adult mice) accompanied by upregulation of several other calcium and magnesium transport systems, including Trpv5, Trpm6, calbindin-D9k, Cnnm2, and Atp13a4. In addition, these animals did not develop nephrocalcinosis and kidney failure, suggesting that there may be compensatory mechanisms in this model that cause calcium and magnesium homeostasis in the absence of claudin-16 [9].

The expression of other claudin isoforms in the kidney has been described, but their role in the control of the selectivity and permeability of divalent cations in the kidney remains unclear [24, 25]. Apparently, the depletion of either claudin-16 or claudin-19 does not affect the expression and localization of other claudins such as claudin-10 and claudin-18, and of other normal constituents of TAL tight junctions, such as occludin and zonulla occludens-1 [26]. Therefore, it is likely that other proteins are involved in the modulation of disease progression or contribute to the differences in the phenotype and biochemical features among FHHNC patients.

The hypothesis of the influence of modulators and epigenetic factors in the clinical spectrum of FHHNC is reinforced by studies that report unusual clinical findings in FHHNC patients with *CLDN16* mutations [27–29] and different clinical courses in siblings with the same *CLDN16* mutation [17, 30]. One case report presented a boy with a truncating mutation in claudin-16 (p.W237X) with early-onset renal insufficiency, horseshoe kidney, neonatal teeth, atypical face, cardiac abnormalities, umbilical hernia, and hypertrichosis [27]. Another study reported a female patient who experienced recurrent passages of kidney stones and urinary tract infections as of 4 years old, and only developed nephrocalcinosis when she was 19 [28]. In another case, genitourinary abnormalities (hypospadias and cryptorchidism) were observed in one of the proband’s siblings [29]. A rare case report described a patient who, in addition to the classical symptoms of FHHNC, was diagnosed with smaller kidneys, severe bone disease, severe metabolic acidosis, and persistent hypocalcemia. Intriguingly, several siblings within the family had died previously without a clear diagnosis [30]. Therefore, there is current evidence that FHHNC may present great variability among patients, with respect to both the clinical manifestation and the genotype-phenotype correlation. The reason that our patient only showed the first symptoms at the age of 20 years old remains unknown, and the role of compensatory mechanisms, epigenetic effects, or other factors merits further investigation.

## Conclusions

Although mutations in *CLDN16* that induce a complete loss of function seem to usually appear early in infancy and progress to CKD at puberty, the phenotype-genotype correlation is not yet completely established. In our patient, the late onset of CKD followed by rapid decline of renal function suggest that further studies are necessary to better explain the differences observed in the clinical course of this disease among patients. This study reports, for the first time, a case of FHHNC due to a multi-exon deletion (E2_E5del) in the *CLDN16* gene, and contributes to improving the phenotype-genotype characterization in these patients.

## Consent

Written informed consent was obtained from the patient and her mother for publication of this case report. There are no accompanying images. A copy of the written consent is available for review by the Editor of this journal. The study was approved by the National Committee in Human Experimentation CONEP (process number 1440/2001).

## Abbreviations

Atp13a4: Cation transporting P-type ATPase
CKD: Chronic kidney disease
*CLDN16*: Gene that encodes the tight junction protein claudin-16
*CLDN19*: Gene that encodes the tight junction protein claudin-19
Cnnm2: Cyclin M2
FHHNC: Familial hypomagnesaemia with hypercalciuria and nephrocalcinosis
*KIAA0056*: Gene that encodes for the protein KIAA0056
MLPA: Multiplex ligation-dependent probe amplification
p.A209T: Residue change from alanine to threonine at position 209
p.F232C: Residue change from phenylalanine to cysteine at position 232
p.G191R: Residue change from glycine to arginine at position 191
p.L145P: Residue change from leucine to proline at position 145
p.L151F: Residue change from leucine to phenylalanine at position 151
p.W237X: Truncating mutation of the tryptophan at position 237
PCR: Polymerase cChain rReaction
TAL: Thick ascending limb of the loop of Henle
Trpm6: Transient receptor potential cation channel subfamily M member 6
Trpv5: Transient receptor potential cation channel subfamily V member 5
*VIPR2*: Gene that encodes for the vasoactive intestinal peptide receptor 2
